# Tiny Flies: A Mighty Pest That Threatens Agricultural Productivity—A Case for Next-Generation Control Strategies of Whiteflies

**DOI:** 10.3390/insects12070585

**Published:** 2021-06-28

**Authors:** Sharad Saurabh, Manisha Mishra, Preeti Rai, Rashmi Pandey, Jyoti Singh, Akansha Khare, Meeta Jain, Pradhyumna Kumar Singh

**Affiliations:** 1 Insect Defense Laboratory, Molecular Biology and Biotechnology Division, CSIR-National Botanical Research Institute, 435, Rana Pratap Marg, Lucknow 226001, Uttar Pradesh, India; saurabh.nbri@hotmail.com (S.S.); rai_preeti02@yahoo.co.in (P.R.); singhjyoti078@gmail.com (J.S.); akanshakhare22@gmail.com (A.K.); 2 Developmental Toxicology Division, CSIR-Indian Institute of Toxicology Research, Vishvigyan Bhawan, 31, Mahatma Gandhi Marg, Lucknow 226001, Uttar Pradesh, India; manisha.nbri@gmail.com (M.M.); rashmipandey0212@gmail.com (R.P.); 3 CSIR-Human Resource Development Centre, Academy of Scientific and Innovative Research (AcSIR), (CSIR-HRDC) Campus, Postal Staff College Area, Sector 19, Kamla Nehru Nagar, Ghaziabad 201002, Uttar Pradesh, India; 4 School of Biochemistry, Khandwa Rd., D.A.V.V., Bhawarkuwa, DAVV Takshila Parisar, Indore 452001, Madhya Pradesh, India; meetajainind@yahoo.com

**Keywords:** whiteflies, *Bemisia tabaci*, RNA interference, target genes, anti-whitefly proteins, genetic engineering, nanotechnology, genome editing, next-generation strategies, viral disease

## Abstract

**Simple Summary:**

Despite being a pest of global importance, effective management of whiteflies by the implication of environmentally friendly approaches is still a far-reaching task. In this review, we have tried to bring the readers’ attention to next-generation control strategies such as RNA interference and genetic modifications of plants for the expression of anti-whitefly proteins. These strategies offer huge promise to provide an effective and sustainable solution to the problem of whiteflies, either in isolation or in combination with other widely used practices under the regimes of integrated pest management. Focus has also been given to advanced technologies such as nanotechnology and genome editing, with promising prospects for field applications. The importance, applicability, and demand of these technologies for the control of whiteflies have been highlighted. We have also attempted to present the holistic picture of challenges in the path of commercial application of these promising technologies. To underline the pest status of whiteflies concisely, we have enlisted all economically important species of the pest along with their host plants/crops across the world. A comprehensive list of various insecticides of chemical, microbial, and botanical origin, applied in the field for the control of sweetpotato whitefly along with their resistance status, ecotoxicities, and effects on biological control agents, has been provided for readers.

**Abstract:**

Whiteflies are a group of universally occurring insects that are considered to be a serious pest in their own way for causing both direct and indirect damages to crops. A few of them serve as vectors of plant viruses that are detrimental to the crop in question and cause an actual loss in productivity. A lot of attention is focused on pest control measures under the umbrella of IPM. In this review, we attempt to summarize the existing literature on how and why whiteflies are a serious concern for agriculture and society. We reviewed why there could be a need for fresh insight into the ways and means with which the pest can be combated. Here, we have emphasized next-generation strategies based on macromolecules, i.e., RNA interference and genetic engineering (for the expression of anti-whitefly proteins), as these strategies possess the greatest scope for research and improvement in the future. Recent scientific efforts based on nanotechnology and genome editing, which seem to offer great potential for whitefly/crop pest control, have been discussed. Comprehensive apprehensions related to obstacles in the path of taking lab-ready technologies into the farmers’ field have also been highlighted. Although the use of RNAi, GM crops, nanotechnologies, for the control of whiteflies needs to be evaluated in the field, there is an emerging range of possible applications with promising prospects for the control of these tiny flies that are mighty pests.

## 1. Introduction

Adoption of *Bacillus thuringiensis* (*Bt)* crops not only transformed the cultivation profile but also altered the pest status as there has been a decline in the prominence of lepidopteran pests and an upsurge of sap feeders such as whiteflies [1]. Some other reasons and factors have also influenced the rise in sap-sucking pests such as declining use of broad-spectrum chemical insecticides [2], either due to environmental concerns or due to redundancies caused by *Bt*-technology; changes in global temperature and humidity patterns that have become more favorable for whiteflies and other sap-sucking pests to thrive [3]. Moreover, chemical insecticides were collaterally able to keep the level of these pests under the economic threshold by virtue of their broad-spectrum nature, and minimization of their usage concomitantly allowed an increase in these sap-sucking pests [1]. Among phloem sap feeders, whiteflies have arisen as a global pest of agriculture, horticulture, and ornamental crops in the last two decades. The presence of whiteflies has been recorded from all continents except Antarctica. It is believed that the most economically important whitefly viz; *Bemisia tabaci* (also referred to as Cotton, Tobacco, Sweetpotato, or Silverleaf whitefly) has made its geographical spread via international transport of infested plant products. Once adopted, it quickly spreads and now it is included among 100 of the “World’s Worst” invaders as per the Global Invasive Species Database (http://www.iucngisd.org/gisd/100_worst.php, accessed on 8 October 2020).

Whiteflies cause up to 100% yield losses due to their direct feeding action, other pathogens that their feeding behavior attracts, and due to the fact that they vector several plant viruses that in turn cause great loss in yields or otherwise severely damage the crops [4]. That these are recognized to be a serious pest can be judged from the extensive economic losses for several crops worldwide, and financial estimates have worked out these losses to be in billions of currency units. It is noteworthy that reliable estimates of the economic impact on global agriculture have not been available in recent years. It might be because it has affected almost all known crops as well as ornamental plants in widespread areas. The concerns deepen when one considers the increasing costs for controlling this pest, which, at the same time, also depreciates the quality of agricultural produces, thereby severely encroaching upon the profitability of the crop production worldwide.

Knowledge about the biological and ecological factors governing pest endurance is a prerequisite for proficient control. Being a multiple-crop pest, whiteflies have numerous hosts and a high reproductive rate which deliver optimal conditions for their expansion. Hence, the development of sustainable management strategies needs a mechanistic acquaintance of factors that affect the growth of the pest population on the montage of host crops and others. Numerous control strategies under the umbrella of IPM, including physical barriers/cultural control measures, biotic agents, pesticides, and host–plant resistance, have been used to combat hemipteran pests in general and whiteflies in particular [5,6]. The IPM strategies mainly focus on keeping the number of adults on plants below an economic threshold level largely by the application of non-eco-friendly chemical pesticides [7]. Widely used insecticides with reported efficacy against the tobacco whitefly, their ecotoxicities, and resistance status in the pest are provided in Table 1. Furthermore, these insecticides are not always whitefly centric and are invariably broad-based, thereby causing collateral damage to beneficial insects that also adds to the economic losses for crop productivity. Insecticide application can manage the whitefly population a bit but causes serious environmental damages in many ways. A dose of chemicals beyond the saturation level might also pose harm to birds and even aquatic organisms (Table 1). Induced resistance in field crop pests including whiteflies (Table 1) under pesticide stress is now considered a universal problem; thus, banking on insecticide-based management is questioned.

These concerns have encouraged us to devise sustainable, specific, and environmentally friendly strategies developed by high throughput scientific intervention. These strategies encompass a combination of practices that specifically targets nymphal stages, life, and fecundity of adults to provide a suitable method for the control of this pest. In the present review, we have discussed next-generation strategies based on macromolecules that offer effective control of this dreadful pest. We have also incorporated some new technologies, e.g., nanoscience and genome editing approaches that are being exploited and appear to have great potential to overcome futuristic challenges. An attempt to highlight the comprehensive picture of various obstacles or challenges on the road to commercialization of these strategies has also been made in this review. Considering that the established strategies by themselves or collectively do not always provide the most viable economic option for the control of whiteflies, we propose some different and/or new approaches for their control, thereby justifying a need for greater insight into the problem that the whiteflies represent.

## 2. A Threat to Agriculture

Whiteflies have a wide range of host plant species, e.g., *B. tabaci* is reported to infest over 900 host plants. A few whiteflies transmit more than a hundred species of plant infecting viruses (that belong to genus *Begomovirus*, *Carlavirus*, *Crinivirus*, *Ipomovirus*, *Torradovirus*), e.g., *Aleurodicus disperses*, *B. tabaci*, *B. afer*, *Trialeuroides vaporariorum*, *T. abutiloneus* and *T. ricini* [4,27,28,29,30]. Some economically important whiteflies from all over the world are summarized in Table 2. The table clearly illustrates that the pest can infest almost all plant species/crops. Knowing the fact that *B. tabaci* can tolerate the long-term high-temperature stress, the menace of whiteflies seems to be aggravated with an increase in average global temperature. It is beyond the scope of this review to enumerate and discuss in detail the manifestation of the pest infestations on all hosts. To highlight the whitefly-inflicted threat to agriculture we are, therefore, considering the examples of a few major cash crops viz; cotton, cucurbits, and tomato where either the economic importance of the crop is high, or the volume of literature reported on whitefly infestations are numerous. In many cases, while the pest infestation per se has not been reported, it is, however, indirectly inferred from the severity and extent of viral disease in the crop that is almost exclusively attributed to whiteflies as vectors for the transmission of viruses. Table 3 represents periodic global incidences of whitefly infestations as well as serious outbreaks and/or reports of plant diseases vectored by whiteflies on important crops in the last 20 years.

The impact of whiteflies on agriculture has far-reaching implications. To illustrate it, we have taken examples of two major cash crops (cotton and tomato) that are severely infested by whiteflies. During the year 2015 in India, cotton grown over approximately 0.58 million ha. in Haryana and 0.4 million ha in Punjab was severely affected by the sweetpotato whitefly, which not only posed pecuniary difficulties (cotton yield loss of ~ 35% worth US$ 630–670 million) but also costed the lives of farmers [76,77]. Tomato is an important constituent of the diet in countries adjacent to the Mediterranean Sea, Sub-Saharan Africa, the Caribbean Islands, Mexico, Central America, Central, South-East Asia, etc. Unfortunately, there are fields of smaller sizes, and families are mainly owned or managed by females and children. Their income is substantially dependent on tomato agriculture, which is usually threatened by tomato yellow leaf curl viral disease. The disease can abolish an entire tomato farm, production in infected fields is often nil and the profit is lost, if not managed. Families are usually left with the option of pesticide application to control the disease and its vector, sometimes daily. This, in turn, causes a financial load on them, health-associated risks to the family members, and pollutes the environment (https://www.cabi.org/isc/datasheet/55402#toimpactSocial, accessed on 12 March 2021). Though the official data about losses incurred by *Tomato Yellow Leaf Curl Virus* (TYLCV) in tomato are not available, the figures are assumed to be in tens of millions of dollars, as quite a few studies report up to 100% disease incidences (Table 3) and yield loss [106]. Notably, the presence of TYLCV in the field is always associated with the presence of *B. tabaci* population across the world. There is a negative correlation between percent disease incidence and number of fruits per plant or total produce in a field [107]. The magnitude of economic losses caused by or attributed to whiteflies can also be judged from socio-economic impact assessments based on scientific efforts. A study based on economic productivity and profitability analysis conducted in the Southeast USA, 2017 described an average return of US$ 1958/ac to produce tomatoes in the presence of whiteflies and TYLCV. The study also calculated the chance to obtain this return to be 50% only [106]. It has also been reported that for every million dollars of *B. tabaci*-induced crop loss in a multi-commodity-growing agriculture community, there was an estimated loss of US$ 1.2 million in the associated sector and unemployment in food processing industries [108]. On the other hand, when crop varieties resistant to whitefly vectored viral disease were used, farmers could gain 10-fold more profit as compared to varieties that are susceptible to viral disease. The adoption of a viral disease-resistant crop was also associated with reduced pesticide uses in the field. It eventually resulted in extra income which increased the livelihood status of farmers in terms of children’s education, better nutrition, and medical expenses [109]. Whiteflies are a crucial pest and require intensive efforts for their control. Despite such efforts and several strategies to control whiteflies, success has been elusive or, at best, sporadic, leading to a growing consensus worldwide that there is a need for a holistic solution to the problem of whiteflies.

## 3. Next-Generation Strategies for the Effective Control of Whiteflies

Combinatorial strategies mentioned above have witnessed limited success; furthermore, these methods largely depend on chemical pesticides, the hazards of which are well documented. It has therefore been imperative to explore alternative approaches that can augment the boundaries of control strategies employed to combat whiteflies. Genetic engineering offers a potential range of solutions to develop transgenic plants harboring desirable traits. It has been used to develop GM crops that are resistant to the pest and associated viral diseases expressing small RNAs, including micro-RNA (miRNA) and small interfering RNA (siRNA) targeting vital genes of different whitefly vectored viruses and the pest. Whitefly-resistant GM crops have also been developed expressing genes encoding insecticidal proteins. It is noteworthy that the selection of vital genes in whiteflies that can be targeted through RNAi and the exploration of anti-whitefly function in the existing battery of insecticidal proteins, along with the discovery of new insecticidal proteins, are the bottleneck in the genetic manipulation approach. A limited number of studies have been performed for the sake of specific biomolecules (including insecticidal proteins) and gene targets (for silencing) that not only have detrimental impacts on whitefly but also have the potential to control whiteflies [110,111]. In this section, we have summarized the studies focusing on the testing of known macromolecules (gene targets for RNA interference and insecticidal proteins), their putative mode of action, and efficacy for the control of whiteflies.

### 3.1. RNAi-Mediated Control

RNAi is the method of silencing the gene(s) or gene families using target gene-specific double-stranded RNA sequences. This technology must go a long way to narrow the gap in agriculture through the production of disease/toxin-free, insect/virus-resistant, and nutritionally rich crop plants. Towards the RNAi-mediated control of the pest, preliminary results are promising to varying degrees [112,113]. The effective implementation of this technology for the control of whitefly is dependent on the presence of siRNA machinery in it [114]. In general, the selection of target gene(s) and the method for the delivery of dsRNA are the two crucial steps for the successful demonstration of effective gene silencing. RNAi for the control of *B. tabaci* through oral route has been demonstrated using an artificial diet [112]. This study has made RNAi feasible for the field application. RNAi-mediated control of whitefly has also been validated in transgenic tobacco wherein a high level of resistance was achieved against the pest in laboratory conditions [113]. Transcriptome and genome sequencing data may be used for the selection of target genes [115,116,117,118,119]. Nevertheless, the identification of whitefly specific genes with no or minimum off-target effects has been a major concern. Therefore, with the help of proteome data, a sincere effort has also been made in the search for sweetpotato-whitefly-specific nucleotide sequences [110].

As already discussed, that the threat of whiteflies is chiefly because of its ability to vector viral diseases in addition to the direct damage, studies (based on RNAi) largely have focused either on controlling the vector or securing protection against whitefly vectored viral diseases. Various metabolic pathways of the whitefly have been targeted for gene silencing and subsequent disruption of the gene product along with its function. Table 4 represents all target genes from several metabolic pathways of the sweetpotato whitefly including their impact and efficacies that have been evaluated for control. Among them, genes involved in energy metabolism, detoxification, cellular transport and osmoregulation, defense, and metamorphosis have gained the maximum attention of researchers around the globe [120,121,122,123]. The silencing of genes involved in the regulation of energy metabolism, cellular transport, and osmoregulation has shown mortality of the pest (Table 4). Similarly, silencing of genes related to defense and immunity has led to compromised fitness and the ability of the pest to cope up with even very low doses of insecticides (Table 4). These targets can be taken up for a detailed study to evaluate their field performance and might be useful in IPM along with other pest control practices. Alternatively, the population build-up of whitefly may be controlled by targeting genes that are majorly involved in embryogenesis and reproduction. Several outcomes, i.e., distortion in egg structure, poor egg filling, reduced number of eggs, and egg sterility, have been observed upon silencing of these genes (Table 4). Disruption in the expression of genes that play a vital role in cell division and intracellular trafficking has shown a range of phenotypic abnormalities in the developmental stages and adults (Table 4). It is important to note that all these genes are neither specific to whiteflies nor cause sufficient toxicity to make them suitable for field application. Therefore, it is crucial to explore other genes in the said pathways for the selection of RNAi targets with enhanced toxicity and specificity.

To achieve protection against *B. tabaci*-transmitted viral diseases, strategies based on pathogen-derived resistance have also been investigated and various detrimental impacts on target viral species have been observed. Targets explored for RNAi ranged from viral genes encoding coat protein, movement proteins to replication-associated proteins, and whitefly genes that play an important role in virus transmission. Notable examples include genes encoding cyclophilin B, HSP70, odorant-binding proteins, GroEL, etc. (Table 4). Silencing of these genes has led to a significant fall in the virus transmission ability of the pest. Some notable viral species include *Cotton Leaf Curl Virus* (CLCuV), *Cassava Mosaic Virus* (CMV), TYLCV, *Tomato leaf curl virus* (ToLCV), *Bean golden mosaic virus* (BGMV), etc. In this section, we have discussed all important studies published to date using RNAi to target viral diseases. Transgenic plants expressing antisense strand of the viral movement protein, “AV2”, and overexpressing truncated replicase gene have been shown to exhibit arrest of the CLCuV infection and resistance, respectively [144,145]. Modified miRNA designed to retain the native miRNA backbone (miRNA 169a) of cotton that contained selected viral (CLCuV) sequences has also been used for the control of the virus. Although, it counters the infection but does not lead to immunity in transgenic plants [146]. Likewise, transgenic cassava lines targeting the replication processes of CMV are found to offer a reduction in the accumulation of viral DNA particles [147]. The RNAi against viral common regions, promoter sequences, and replication-associated protein-coding sequences has been successful in combating very high virus loads up to 98% [148,149,150]. Resistance against TYLCV has been successfully demonstrated in transgenic tomato plants using antisense technology against replication-associated coding sequence [151]. Similarly, the transgenic tomato-harboring coat protein gene of ToLCV has been shown to offer a variable degree of disease tolerance [152]. The IL-60 system derived from TYLCV for silencing in plants has also been used to develop resistance/tolerance against TYLCV [153]. The transgenic approach employing the RNAi against the viral “*AC1*” gene to control BGMV has been highly successful and commercialized in Brazil. GM plants have shown high resistance and absence of symptoms in more than 90% of the plants [154,155]. However, transgenic bean plants expressing the mutated *rep* gene have shown only partial resistance against BGMV [156]. Though successful to varying degrees in conferring resistance to whitefly vectored viral diseases, these transgenic approaches are, however, restricted to the disease per se. What is expected in the transgenic approaches for whitefly control is prevention and/or elimination of the pest infestation since this may offer a generic and widely applicable strategy for control.

### 3.2. Anti-Whitefly Proteins

Advancements in genetic engineering technologies, as well as knowledge of the bacterial insecticidal toxins, have led to the development of *Bt*-insecticidal toxin-based crop protection strategy especially against lepidopteran pests, where transgenics producing engineered *Bt* toxin has been used. Hence, the expression of transgenes with reported insecticidal activity from microorganisms, plants, and animals has been explored for control of the sweetpotato whitefly. Recently, Liu et al., 2020, have reported toxicity (LC_50_ = 15.57 μg/mL in insect bioassay using artificial diet) of a protein elicitor viz; AMEP412 from *B. subtilis* against *B. tabaci* [157]. In silico study has revealed that the hydrophobic peptide of AMEP412 is critical for the mortality of whitefly as it shows features of pore formation by interacting with cell membranes and causes cell lysis. However, the potential of toxin(s) from *Bacillus* sps. needs to be proven. Likewise, lectins of plant origin such as *Allium sativum* leaf agglutinin (ASAL; LC_50_ = 8.5 μg/mL) from *Allium* (garlic) leaves, *Pinellia ternata* agglutinin (PTA) from *Pinellia ternate* (Chinese medicinal herb), *Remusatia vivipara* lectin (RVL1) from *Remusatia vivipara*, and *Colocasia esculenta* agglutinin (CEA; LC_50_ = 5.17 μg/mL in insect bioassay using artificial diet) from *Colocasia esculenta* (taro) have been reported to confer toxicity against the sweetpotato whitefly [158,159,160,161]. Transgenic plants expressing ASAL, and PTA have been found to exhibit various entomotoxic effects with low mortality such as decreased nymphal emergence, development, fecundity, and population build-up of *B. tabaci* [158,159]. Application of lectins in genetic engineering approaches might be limited due to their higher doses required to obtain significant control over the pest and prevention of viral transmission. To enhance the toxicity of lectins against the sweetpotato whitefly, a mannose-binding lectin, namely, *Galanthus nivelis* agglutinin (GNA) from *Galanthus nivelis* (Snowdrop), has been fused at the C-terminus of a neurotoxic peptide from a scorpion and engineered in tobacco. The transgenic tobacco plants expressing fusion protein have shown increased toxicity in *B. tabaci* as a decreased number of nymphs, and reduced egg hatching rate has been observed [162]. It has suggested the potential use of plant lectins as carrier molecules for the anti-whitefly proteins to achieve targeted control. Another class of insecticidal proteins that has been explored against whiteflies is “chitinases”. Although entomopathogenic fungi, e.g., *Metarhizium* sps., *Isaria fumosorosea*, etc., producing chitinase is an effective biological control agent for whiteflies [163], very few chitinases are found to be toxic against whiteflies [164,165]. An organized and efficient exploration of fern biodiversity has been performed in want of anti-whitefly protein(s) and a protein viz., Tma12 (LC_50_ = 1.49 μg/mL in insect bioassay using artificial diet) has been discovered [111]. Tma12 is a lytic polysaccharide monooxygenase and is the first LPMO from the plant [166]. It interferes with the reproductive biology of whitefly in GM cotton and hence provides excellent control over the pest. It is noteworthy that GM-cotton-expressing Tma12 is also found protected from cotton leaf curl viral disease [111]. In addition, approaches to manipulate the host plant’s metabolite profile have also been used. Broad-spectrum resistance against sap-sucking pests including whitefly has been achieved by increasing the methanol content in transgenic tobacco plants [167].

There is tremendous scope for synthetic biology, as well as miRNA/siRNA technology, to be developed with high specificity and efficacy against whiteflies. Genome sequences of whiteflies may serve as good sources for the development of new control strategies. In another potentially exciting and innovative approach, plant proteins that are specifically toxic to whiteflies should be explored and the genes encoding such proteins can be engineered in such a way that the greatest expression is achieved in phloem cells or at least the engineered and expressed protein is preferentially loaded in the phloem to exert its toxic effect on whiteflies. Such a pest-centric approach will not only be generically applicable on a wide class of whiteflies but perhaps be also crop and cropping region independent in its scope and application.

## 4. Futuristic Strategies for the Management of Whiteflies

Though genetic engineering offers markedly improved protection against the whitefly, there is a scope to adopt new or path-breaking techniques/technologies for the control of this pest and to avoid complex deregulatory procedures to grow GM plants in the field. Many countries and a few groups of intellects are not in favor of GM crops. Therefore, researchers are continuously trying to discover some novel molecules and explore new methods/approaches that can lead to a revolution in insect pest control and that may be proven as a milestone. 

### 4.1. Nanotechnology: A Bliss for Crop Protection

Nanotechnology has emerged as a revolutionary technology in the field of agriculture including pest control in the past two decades. The utilization of nanoscience in plant protection via nanoparticles/nanomaterial-based pesticides has attracted a lot of research interest. These materials are taking preference over chemical pesticides because they offer higher surface area due to their nano size and thus quicker action. The unique physical and chemical properties of nanosized particles make them different from their macro-scale counterpart that in turn enhance their applicability in pest management programs. Their application might minimize the negative impacts posed by chemical pesticides. Various synthetic- and bio-nanomaterials (nano-emulsions, -particles, -fibers, -tubes, -spheres, -sheets, -onions, graphene, and their derivatives, etc.) are being explored for the control of several crop insect pests, including sap-sucking pests such as aphids, and whitefly. The potential of nanoparticles is largely tested in two different ways (i) nanomaterials themselves used as pesticides [168,169], (ii) as additives to enhance the toxicity of currently available natural/synthetic insecticidal oil and formulations, etc. [170]. A literature survey reveals that the management of whitefly through the application of nanomaterials is in its nascent stage. In this review, we have discussed all studies focused on nanomaterial-based control of sap-sucking pests, as the whitefly possesses piercing and sucking type of mouthparts and shares similar feeding habits. 

Nanomaterials have been a potent candidate pesticide (nanopesticides) for pest management. In a laboratory choice bioassay, nano-emulsions prepared from essential oils and pure compounds have shown a strong repellency function against the bird cherry-oat aphid, a major pest of cereal crops in temperate regions globally. Authors have reported repellency index values ranging from 68.8 to 100 using farnesol, geraniol, cis-jasmone, etc. [171]. Nanotization of a chemical pesticide viz: deltamethrin has resulted in ~10-fold enhancement in the toxicity of the pesticide against the green-house whitefly (*T. vaporariorum*) [172]. Researchers have also explored metal-NPs in general and Ag- and Zn-NPs in particular as pesticides to achieve effective control of sap feeders [173,174,175]. For example, Bhattacharya et al., 2016, synthesized biologically active Ag-NPs from tomatoes and tested against the rose aphid, *Macrosiphum rosae* (Hemiptera: Aphididae), a key pest on the rose plant. When leaves were dipped in insecticidal solution of Ag-NPs at a concentration of 400 and 500 ppm, 100% mortality of adults was observed in four and three days, respectively [173]. Similarly, decreased population density of *B. tabaci* nymphs was recorded on leaves of eggplants treated with Ag-NPs (3000 ppm) prepared from jujube leaf aqueous extract under greenhouse conditions [174]. Rouhani et al., 2011, assessed the insecticidal activity of Ag- and Ag-Zn nanoparticles on the 1 d old first instar nymphs of oleander aphid (*Aphis nerii*) and found the LC_50_ values to be 424.67 mg/mL and 539.46 mg/mL, respectively, after 24 h of treatment. They reported a significant increase in mortality with the increase in the concentration of nanoparticles [175]. Another widely used NP is zinc oxide (ZnO), which is commonly used as a fungicide in agriculture. Khooshe-Bast et al., 2016, analyzed the insecticidal activity of ZnO-NPs on greenhouse whiteflies under laboratory conditions. Synthetic ZnO-NPs had significant lethal impact (91.6% mortality in 24 h after treatment) on adults (LC_50_ = 7.35 mg/L) in a concentration-dependent manner [176]. A combination of three metal-NPs viz: TiO_2_, ZnO, and Ag were also shown to have insecticidal activity (LD_50_ = 195.27mg/L) on western flower thrips, *Frankliniella occidentalis* [177]. Authors also showed that the mortality to the pest pertained maximum to TiO_2_- (70%) followed by ZnO- (28%) and Ag- (2%) NPs. When the potential of NPs was compared with that of commonly used insecticides, the latter was found to be more potent. For example, the LC_50_ value for imidacloprid after 24 h of treatment against oleander aphids (0.13 μL/mL) was estimated to be slightly lower than that of Ag- (424.67 mg/mL) and Ag-Zn NPs (539.46 mg/mL), respectively [175]. Similarly, Samih et al., 2011, compared the efficacy of ZnO, and ZnAl_2_O_3_ with the Amitraz (a well-known insecticide) against pistachio psylly (*Agonoscena pistaciae*), one of the most detrimental pests to pistachio trees. The insecticide was found to have a greater insecticidal impact over tested NPs [178]. Yet, one obvious advantage of using NPs is the low risk of resistance development against them as they have a slower effect than the chemical pesticides used in the field. These studies advocate the use of NPs for pest management instead of using harmful chemical pesticides to minimize the risk of resistance development as well as harmful effects on the environment.

Further being used as pesticides, a range of NPs have been tested for the fold enhancement in the bioactivities of traditional insecticides of botanical origin (e.g., essential oils) and chemical pesticides. Potential use of NPs as effector on biological control agents/microbial formulations/extracts have also been explored. With this approach, the cumulative protection of crop plants has reached the next level. Christofoli et al., 2015, have shown increased efficacy (95% reduction in fecundity) of essential oils (B-elemene, alphaelemene, B-caryophyllene, D-geracrene) isolated from *Zanthoxylum rhoifolium*, when encapsulated with poly € caprolactone nanosphere and spread over tomato leaves as compared to its application alone, which exhibited 82% reduction in fecundity of *B. tabaci* [179]. These studies have demonstrated how particle size impacts the biological activity of a given molecule. Furthermore, zero-valent iron NPs have been shown to enhance the *Isaria fumosorosea*-mediated biological control of *B. tabaci* as increased larval mortality and reduced egg hatchability have been observed [180]. The raw carbon NPs (biochar) have also been found to enhance the entomo-pathogenicity of *Cordyceps fumosorosea* on *B. tabaci*. The 100-ppm conidial suspension of fungi has led to 50% nymphal mortality; however, when fungal suspension has been applied with biochar, 90–100% mortality has been observed at a 2-fold lower concentration [181]. Slow and steady release of agrochemicals such as insecticides, biological control agents, formulations, etc., with the help of nanoparticles that are also known as controlled release formulations (CRFs) has been shown to favor the control of pests in the field for a longer duration [182]. RNAi-based systemic protection has been demonstrated against *Cucumber Mosaic Virus* through the sustainable release of dsRNA, carried by a designer, non-toxic, degradable, layered double hydroxide (LDH) nanosheets. LDH-dsRNA complex was referred to as BioClay. Topical application of BioClay has protected leaves challenged with the virus even after 20 days of spray [183]. These approaches also provide a way to prevent the toxic/active molecules from being photodegraded and maximize the effects even at a low concentration of the active principle. 

NPs can penetrate the cuticle, cell, plasma membrane and cause breakdown/coagulation of proteins/enzymes or the plasma membrane to lose its stability/function, which eventually leads to the death of a cell. In general, the nanomaterial-mediated toxicity can be explained by alterations in the basic physiological activities in the insect, e.g., molting or by activating the reactive oxygen species, hence, oxidative stress in insects [184], which in turn resulted in a range of larvicidal/insecticidal activities. In conclusion, the application of NPs may lead to a delay in pest-resistance mechanisms to chemical insecticides. It may offer economical, as well as eco-friendly, ways for long-term usages in the future to achieve crop protection even at a lower concentration of insecticide if the application of insecticide is unavoidable. Nanotechnology may also be utilized to enhance the efficacy of known molecules with anti-whitefly function. Notably, all the studies have been conducted in laboratory conditions; therefore, the field application of NPs for the control of pests requires serious scientific efforts. 

### 4.2. Genome Editing

After the discovery of specific endonucleases (meganuclease, zinc finger nuclease, and transcriptional activator-like effector nuclease) and the CRISPR/Cas system, the usefulness of the genome editing approach for various applications of human importance came into the picture. In an excellent example of pest control based on genome editing, the wild population of *Aedes* mosquito has been significantly reduced in Brazil, Panama, and Cayman Islands (https://cogem.net/app/uploads/2019/07/CGM180501-01-CRISPR-Animals-Implications-Genome-Editing-2018_HR1.pdf, accessed on 8 November 2020). Although, genome editing for the control of crop pests has not been explored to date, researchers have successfully established the protocol for editing of target genes in crop pests, e.g., Egyptian cotton leafworm (*S. littoralis*), pine caterpillar moth (*Dendrolimus punctatus*), citrus psyllid (*Diaphorina citri*), and sweet potato whitefly. Koutroumpa et al., 2016, knocked out the *Orco* gene encoding olfactory receptor co-receptor in *S. littoralis* and reported the impairment in pests’ ability to detect the plant odor and sex pheromone in homozygous individuals. The success rate of mutations was found to be 89.6% (injected individuals carried *Orco* mutations) [185]. Liu et al., 2017, introduced the CRISPR/Cas9 system in the pine caterpillar moth, a devastating forest pest to manipulate the expression of *Wnt-1* gene, which is associated with early body planning of the moth. The mutation rate was relatively less as only 32.9% of embryos and larvae exhibited abnormal development, loss of limbs, and head deformity [186]. Targeted mutagenesis based on microinjection of fertilized eggs using the CRISPR/Cas9 system was demonstrated in pea aphids (*Acyrthosiphon pisum*). Authors targeted the gene *stylin-01* encoding a cuticular protein and reported the 70–80% mutation rate in eggs. However, only 1–11% of injected eggs could be hatched and ~35% mutation rate could be observed in the germline [187]. Hunter et al., 2018, established a protocol for heritable germline gene editing in Asian citrus psyllid (*Diaphorina citri*), a devastating pest of citrus industries in the USA [188]. The Branched Amphiphilic Peptide Capsule (BAPC)-assisted CRISPR/Cas9 system enabled delivery directly into nymphs and adult females through injection of CRISPR/Cas9 components near ovaries. It was a breakthrough method which evaded the requirement of microinjections in eggs and made the gene editing possible across arthropods. It is noteworthy that earlier attempts to deliver CRISPR components via embryonic injections have largely been unsuccessful due to nearly complete mortalities in embryos, especially in hemipterans. Very recently, genome editing protocol in *B. tabaci* has been developed by an international team of researchers. Authors have shown successful injection of this tiny pest with the ovary targeting peptide ligand fused with Cas9 resulting in heritable editing of the genome in the progeny [189].

Editing of crop plants for insect/pathogen resistance is also at its nascent stage. Nevertheless, researchers have targeted important genes of metabolic pathways that are involved in the production of volatile blends. It is one of the biggest factors of plants that can decide to attract or deter the insect pests upon them. The plant-originated pheromone blends are known for their role in the growth, immunity, and behavior of insects. In a study, CRISPR/Cas9-based knockout of the cytochrome P450 gene, namely, CYP71A1 (CYP71A1-KO) was created in the rice model. The gene encodes an enzyme tryptamine 5-hydroxylase, which catalyzes the conversion of tryptamine to serotonin in rice. Knockout mutants exhibited a significant reduction in growth and development of brown planthopper (*Nilaparvata lugens*) and striped stem borer (*Chilo suppressalis*), the two most destructive pests of rice. It was due to the reduced biosynthesis of serotonin which is essential for the larval growth and development in pests [190,191]. Recently, Zhang et al., 2019, have reported efficient genome editing in soybeans using the CRISPR/ Cas9 mediated multiplex gene-editing system to achieve protection against *Soybean Mosaic Virus* vectored by aphids. Authors have simultaneously targeted three genes of the isoflavone pathway, namely, *GmF3H1, GmF3H2* (*Glycine max flavanone-3-hydroxylase*; F3H), and *GmFNSII-1* (*flavone synthase II; FNS II*) in soya bean hairy roots, and plants. They have reported higher triple gene mutation efficiency (44.44%) in transgenic plants with stable inheritance in progenies as compared to hairy roots. Triple mutants in T3 generation have exhibited a two-fold increase in isoflavone content in leaves and a significant reduction in coat protein content of the virus. Increased isoflavone content acts as an antagonist for virus binding, replication, multiplication, and protein translation and eventually has conferred enhanced resistance to *SMV* in leaves [192]. Soon after its introduction as a new breeding methodology, genome-editing approaches, continuously gaining ample research interests. As the edited organism would not contain any exogenous/foreign DNA or transgenes, they are similar to those developed from traditional breeding and stock-development approaches. These added advantages make genome editing technology superior over other genetic manipulation approaches and must be excluded from the existing boundaries of regulatory guidelines. Furthermore, the simplicity of this technique enables scientists to explore new routes to get rid of losses offered by the crop herbivores utilizing the genetics of insects and/or target crops. In nutshell, genome-editing technology could pave the way for the development of novel pest control strategies against crop pests including sap-sucking insect pests such as whiteflies, aphids, etc., through interference in their development or other vital biology such as virus transmission capabilities.

## 5. Obstacles/Challenges on the Road to Commercialization: From Laboratory to Field

Scientific interventions leading to new tools and technologies have been playing an important role in improving human lives. In this regard, the potential application of genetic modifications and nanotechnology has been demonstrated in a wide range of sectors, directly or indirectly related to the flora and fauna on the earth. These potential applications include, but are not limited to, improved crop protection by enhancing crop productivity or minimizing the losses imposed by pests and pathogens. Nevertheless, the associated challenges/obstacles such as safety and social acceptance, etc., linked with the development and release of any new technology in the field cannot be overruled. In this section, we have tried to highlight obstacles in the path of taking lab-ready technology into the farmers’ field. 

Technologies developed through genetic engineering are effective, highly demanding, and largely safe. GM crops expressing dsRNA and/or insecticidal proteins have many obstacles that need to be addressed well before their commercial application. For example, GM plants offering RNAi might also have off-target effects besides their impact on specific pests. It raises a major concern for the utilization of this technology. Therefore, sincere efforts are required to identify the most suitable/specific target nucleotide sequences. A major limiting factor with GM-based technologies is their deregulation process, though it varies a lot across the globe. For example, regulatory policies in the USA, Mexico, Canada, Brazil, Bangladesh, etc., are relatively lenient as compared to the UK. However, a GM crop needs to undergo screening to avoid any potential hazards it may possess. In India, genetically engineered products are regulated under biosafety regulatory framework established under “Manufacture, use, import, export, and storage of hazardous microorganisms/genetically engineered organisms or cells, Rules 1989 (Rules 1989) under Environment (Protection) Act (EPA), 1986”. An utmost requirement for any GM-based technology to be implemented in the field is the data of field trials along with the biosafety assessments as per the guidelines prevailing in the country, where its commercial application is anticipated. It is a cumbersome and time-consuming obligatory requirement; hence, generation of deregulatory data within time is the greatest hurdle for GM technologies. 

Genome editing for the control of crop pests has a long way to meet the expectation of growers. Largely, protocols for efficient gene editing in a few crop pests have been established. This technology is under investigation at lab scale for many pests and appears to hold great potential for futuristic pest management strategies. However, it has to achieve many milestones in the lab, as well as in the field, to prove its candidature as potential tactics for pest control. The development of resistance against sex-specific lethal traits in the edited organisms is a foremost risk factor at both theoretical and experimental levels. Some other challenges associated with the technology are as follows: (1) optimization of a best-suited method for the delivery of functional assembly required for genome editing inside the target cell, (2) acceptability and responsiveness of the target cell, (3) controlled release, mating and simple screening method for easy diagnosis of edited genotypes with the desired trait in insects, (4) high possibility of the horizontal gene (trait) transfer between edited species and its genetically related species, and (5) calculation of any post-release risk assessment on non-target and/or beneficial insects, etc. Along with these concerns, genome-edited crops also require approval from regulatory bodies, which of course is another major hurdle. The basic framework of regulatory guidelines for the research and development of genome-edited organisms is similar to those for transgenics; however, architecture stringency varies. The approval requirements depend on the purpose for which approvals are sought, the extent of modification(s) introduced, and the risk levels of the resulting organisms. Genome-edited organisms/products are grouped in three categories based on the level of threat they may pose. Group 1, i.e., genome-edited 1 (GEd1) or Site-Directed Nuclease 1 (SDN1), is referred to as single or a few base-pair edits/deletions/insertions leading to change(s) in genome equivalent to those observed in conventional breeding methods. GEd 2 (SDN2) covers several base pair edits leading to a certain degree of changes in the phenotype/genotype of the target plant leading to the improvement of an existing attribute or creation of a new attribute. Changes in the genome of a plant might result in a gain of function with a new protein or RNA. The commercial application of products/technologies developed through SDN2 requires safety assessment up to a certain extent. GEd 3 (SDN3) denotes insertion of foreign gene/DNA sequence that is equivalent to transgenics and leading to a high degree of changes in genotype and/or phenotype in plants that results in the creation of a new attribute, e.g., changes in metabolic pathway(s), etc. Such changes will cause a gain of function with a new protein or RNA. Genome-edited plants/crops developed through SDN3 are considered transgenic. Hence, their field application requires complete safety data similar to those for transgenics. The intricacy of circuitous deregulation journey is further elevated by the social and economic status of countries. Besides being very useful, only a small number of GM/GE technologies could reach farmers’ fields because of very little public acceptance in many countries. Petite scientific cognizance and prevailing myths such as “*GMOs cause cancer, autism, allergies, and other illness*”, “*if livestock eats GM grains, there will be GMOs in meat, milk, and eggs*”, etc., together with religious belief, i.e., “*Tempering with nature is the only cause of natural calamities*” discourages citizens of any country to accept GM/GE technologies. This also poses a serious challenge for researchers/policymakers/industrialists to adopt these technologies. 

It is evident from the literature search that the NP-based control of crop pests is under investigation. Therefore, we have also presented a glimpse of scientific questions that may be required to answer appropriately before the successful implication of nanoscience-based technology for the control of crop pests in the field. The major question linked to the multi-facet application of NPs is: Are these tiny particles safe for the environment? Nonetheless, it is quite difficult to justify the safety concerns linked to nanoparticles in the absolute terminology of yes or no. Besides being a highly emerging technology with the potential application, the proven and speculated apprehensions of nanotechnology are as follows: (1) lack of well-defined safety measures and guidelines for R&D based on NPs, including the requirement of biosafety data, (2) unavailability of regulatory guidelines for the field application, (3) requirement of protocols/methods/techniques for the detection of NPs in food materials, (4) if present in food, determination of the acceptable (non-detrimental) intake threshold, (5) toxicity to other non-target living beings and, last but not the least, (6) bioaccumulation and risk management, if any.

## 6. Conclusions and Future Perspectives

Whiteflies pose a very serious challenge to crop productivity due to their (1) polyphagous nature, (2) worldwide occurrence (3) biological diversity, and (4) virus transmission capabilities. The pest can infest almost all crops or vegetables as depicted in Table 2 and has developed an inclination to switch to the next available host very quickly when the previous one is harvested. Infestations of whiteflies not only affect the plant per se but also several facets of the economically important end-products. More information is available about whitefly-vectored viral diseases on crops such as tomato, cotton, cucurbits, etc., across the globe. This is perhaps because these are cash crops and their viral diseases have been best studied all over the world. Among indirect damages, the transmission of diseases by some whiteflies is the most important consequence often leading to complete yield loss. The list of countries/territories in Table 3 indicates the preponderance of the pest infestation and disease epidemics to occur in regions where whiteflies are in the greatest abundance, especially the tropical and sub-tropical regions of the world. Various review articles on several whitefly-vectored viral diseases in recent years indicate that these diseases are the foremost threat to crop production. However, based on the fact that these diseases are vectored by whiteflies, it is the whiteflies that must be regarded as the main cause for loss in crop productivity. Control of whiteflies is very challenging as it reproduces very quickly and develops resistance to insecticides. In the present time, the use of insecticides either alone or under the umbrella of IPM is the only and major approach employed by farmers in most of the countries to manage whitefly populations because of their usefulness and convenience. IPM is, however, not foolproof primarily because strategies are specific to a given cropping season, geographical area, as well as the crop in question, and also because it is not economically feasible at all times for various crops or in all countries.

The measures for controlling whiteflies have been falling short of the required high mortality (95–100%) at low concentration, and the complete elimination of whiteflies indicates their inefficiencies. We have witnessed a paradigm shift with which we have approached whitefly control in the last decade. It is largely turning away from harmful pesticides towards more environment friendly and sustainable strategies, e.g., RNAi and genetic engineering approach. These next-generation strategies have the greatest scope for research and improvement in the future. Various vital genes in important metabolic pathways have been targeted through RNAi. Going forward, a careful and critical exploration of the generic region, unique to whiteflies should be performed using the available transcriptome of whiteflies so as to avoid off-target effects, if any. The selected generic region/genes encoding unique proteins would serve as the target for RNAi. Likewise, evaluation of known insecticidal proteins for possible anti-whitefly function has not shown very exciting results. So, the discovery of new whitefly-specific insecticidal protein(s) and their expression in transgenic crops that provide high resistance is desirable because this technology could be provided to end-users as seeds. However, there is no report of such a transgenic crop to date that provides trustworthy and stable resistance to whiteflies. There is a belief that nature is replete with a plethora of metabolites and macromolecules that have the potential to act as a toxin to one class of organisms even as they are beneficial to yet another class. Acting on this belief, exploration of our huge plant biodiversity for a variety of molecules that can be extracted and tested for their efficacy against the whiteflies should be the future approach. Targeted exploration of proteins specific to whiteflies that are toxic at low ppm level and then incorporation of genetic engineering tools for the development of transgenic plants resistant directly to whiteflies may be one of the best strategies to overcome the pest as well as associated diseases. The introduction of *tma12* into cotton has been a major success in terms of the levels of protection offered. The next generation of whitefly-resistant transgenic plants must be designed in such a way that it achieves maximum expression of anti-whitefly proteins in phloem and also prevents the inception of resistance and hence provides sustainable crop protection. We are of the view that these two approaches, where RNAi against a unique generic region of whiteflies or protein(s) specific to whiteflies are involved, would deliver a comprehensive solution to the problem that will be target-specific and relatively safe to the environment as well.

With the development of science and technology, some new whitefly control strategies based on nanotechnology and genome editing have also been tested. Although these approaches are at the budding stage, preliminary results offer great potential for whitefly control in the future. The promising results obtained in laboratories should be further evaluated and validated for their field applications. However, care should be taken that the modification will not lead to deleterious effects on the beneficial insect population. These outstanding technologies should be employed to design whitefly-specific nanoparticles to confer resistance in crops. Moreover, semiochemicals are also being studied for pest management. The discovery of a true repellent of whitefly that prevents the colonization of the pest should be the future target. Active NPs may also be exploited as a carrier for whitefly-specific siRNAs, new anti-whitefly proteins, or volatile organic compounds via sprays or slow-release dispensers. It is also crucial to make the public aware and willing, especially in developing countries, to accept GM/GE crops. This will encourage researchers to generate the safety data required for the deregulation of these technologies and to take the lab-ready technologies in farmers’ fields rather than restricting them to good journals. An open forum discussion among the scientists, policymakers, industrialists, farmers, NGOs, hardliners, etc., should be held to identify a roadmap to overcome all probable issues linked with GM/GE technologies. Countries should also increase their gross domestic product investment in R&D related to whitefly management based on GM/GE crops and/or nanotechnology to promote innovation, in the agriculture sector, where these technologies hold potential for ‘Agriculture Revolution’. These multiple whitefly-centric strategies will ensure a successful campaign towards control of these tiny flies that are in actuality a mighty pest.

## Figures and Tables

**Table 1:** Widely used insecticides with reported efficacy against whiteflies.

Site of Action	Class of Chemical	Example	Resistance Development in *B. tabaci*	Ecotoxicity				Approval for Use/WHO Classification	Effect on *Encarsia* spp. or *Eretmocerus eremicus*	References
				Mammals Acute Oral Toxicity	Birds	Fishes	Bees			
**Chemical Insecticides**										
GABA gated chloride channels	Organo-chlorines	Endosulfan	Yes	H	M	VH	M	NA/II	II *^c^*	[8]
		DTT	Yes	M	PNT	M	M	NA/II	-	-
		Lindane	No	M	M	VH	H	NA/II	IV *^c^*	[9]
	Phenyl- pyrazole (Chlorpyriphos)	Fipronil	Yes	M	H	M	H	A/II	-	-
AChE inhibitors	Organo- phosphates	Malathione	Yes	S	M	H	H	A/III	IV *^c^*	[10]
		Acephate	Yes	S	M	S	M	NA/II	IV *^c,d^*	[10,11]
	Methyl- carbamates	Aldicarb	Yes *^a^*	VH	VH	M	H	NA/Ia	-	[12]
		Carbosulfan	Yes *^a^*	M	H	VH	H	NA/II	-	[12]
Sodium channel inhibitors	Synthetic pyrethroids	Bifenthrin	Yes	H	S	VH	H	A/II	I *^c^*/IV *^d^*	[11,13]
		Fenpropathrin	Yes	S	S	VH	H	NA/II	IV *^d^*	[9]
nAChR agonist	Neonicotinoids	Acetamiprid	Yes	M	M	M	M	A/II	IV *^c,d^*	[10,11]
		Imidacloprid	Yes	M	H	S	H	A/II	III *^c^*/IV *^d^*	[10,11]
	Sulfoximine	Sufoxaflor	No	S	S	S	H	A/NL	-	-
Inhibitors of mitochondrial ATP synthase I	Thiourea	Diafenthiuron	No	S	S	VH	H	NL/III	I *^c^*	[8,14]
Salivary pump inhibitors	Pyridine-azomethines	Pymetrozine	Yes	PNT	PNT	M	PNT	A/NL	I *^c,d^*	[11]
	Pyridinecarboxa-mide	Flonicamid	-	S	PNT	M	PNT	A/NL	-	-
Inhibitors of mitochondrial electron transport complex I	Pyridazin	Pyridaben	Yes *^a^*	M	PNT	VH	H	A/II	IV *^c,d^*	[11,15]
	Pyrazole	Tolfenpyrad	-	M	M	H	-	NL/NL	IV *^c,d^*	[10,11]
Inhibitors of lipid synthesis	Derivatives of Tetronic acid and Tetramic acid	Spiromesifen	Yes *^a^*	PNT	PNT	H	PNT	A/NL	-	[15]
		Spirotetramat	No *^a^*	PNT	PNT	M	PNT	A/III	-	[16]
Ryanodine receptor modulators	Diamides	Cyantranilipro-le	-	PNT	PNT	S	H	P/NL	-	-
		Chlorantranili-prole	-	PNT	PNT	S	M	A/U	I *^d^*	[17]
Unknown	Quinazinalone	Pyrifluquinazon	-	M	S	-	-	NL/NL	-	-
**Insect Growth Regulators**										
Juvenile hormone mimic	JHA	Fenoxycarb	No	PNT	PNT	M	PNT	A/U	III *^d^*	[18]
		Pyriproxyfen	Yes	PNT	S	M	PNT	A/U	I *^c^*/IV *^d^*	[13,18]
		Kinoprene	No	PNT	PNT	S	PNT	NA/O	IV *^d^*	[18]
Chitin synthesis inhibitor type 0	Benzoylureas	Novaluron	No	PNT	PNT	M	PNT	A/U	I *^c^*	[19]
		Teflubenzuron	No	PNT	PNT	VH	PNT	A/U	I *^d^*	[20]
Chitin synthesis inhibitor type 1	Unclassified	Buprofezin	Yes	PNT	PNT	H	PNT	A/III	I *^c^*/III *^d^*	[18,21]
**Insecticides of Microbial Origin**										
Glutamate gated chloride channels	Avermactins Macrocyclic lactone	Abamectin	Yes *^a^*	H *^b^*	S *^b^*	H *^b^*	H *^b^*	A/NL	III *^c^* I *^d^*	[10,13,22] [17,23]
GABA gated chloride channels	Spinosad	Spinosad	-	PNT	PNT	S	H	A/III	III *^c^*	[13,24]
**Insecticides of Botanical Origin**										
Mitochondrial ET complex I inhibitors	Rotenone	Rotenone	-	-	-	-	H	-/II	-	-
Antifeedant and anti-molting	Limonoid	Azadirachtin	Yes *^a^*	S	S	VH	PNT	A/NL	II *^c^*	[13,15,25]
Voltage gated sodium channel blockers	Pyrethrins, Oleoresin	Pyrethrum	-	S	S	H	H	A/II	IV *^c^*	[26]

Note. Resistance development in *Bemisia tabaci* is according to Arthropod Pesticide Resistance Database ^1^, unless otherwise indicated. The data for ecotoxicity have been taken from the International Union of Pure and Applied Chemistry (IUPAC) ^2^, unless specified otherwise. The ecotoxicity classification for terrestrial and aquatic organisms is based on US-Environmental Protection Agency (USEPA) ^3^. The contents in column 6 are taken from USEPA ^4^ and the WHO Recommended Classification of Pesticides by Hazard and Guidelines to Classification 2009 ^5^. The toxicity scale for *Encarsia* spp. or *Eretmocerus eremicus* has been determined from I to IV as mentioned. (-) Data not available. Ecotoxicity data: VH—very high; H—high; M—moderate; S—slightly; PNT—practically non-toxic. Approval for use: NA—not approved; A—approved; P—pending; NL—not listed. WHO classification: I—extremely hazardous; II— moderately hazardous; III—slightly hazardous; O—obsolete substance; U—unlikely to present an acute hazard; NL—not listed. Toxicity scale of parasitoids: 0–24%—I; 25–49%—II; 50–74%—III; 75–100%—IV. AChE: acetylcholinesterase; nAChR: nicotinic acetylcholine receptor; JHA: juvenile hormone antagonist. *^a,b^* Reference for these are given in the table; *^c^* effect on *Encarsia* spp; *^d^* effect on *Eretmocerus eremicus*. ^1^ http://www.pesticideresistance.org/ (accessed on 8 November 2020). ^2^ http://sitem.herts.ac.uk/aeru/iupac/ (accessed on 8 November 2020). ^3^ https://www.epa.gov/pesticide-science-and-assessing-pesticide-risks/technical-overview-ecological-risk-assessment-0 (accessed on 8 November 2020). ^4^ http://iaspub.epa.gov/apex/pesticides/f?p=PPLS:1 (accessed on 8 November 2020). ^5^ http://www.who.int/ipcs/publications/pesticides_hazard_2009.pdf (accessed on 8 November 2020).

**Table 2:** Economically important whiteflies and their host plants.

Scientific Name	Common Name	Important Host Plants/Crops	Reference
*Acaudaleyrodes rachipora*	Babul whitefly	Many arid zone forestry tree species	[31]
*Aleurocanthus arecae*	Arecanut whitefly	Arecanut and coconut	[31]
*Aleurocanthus camelliae*	Camellia spiny whitefly	Tea	[32]
*Aleurocanthus rugosa*	Betelvine whitefly	Betelvine	[31]
*Aleurocanthus spiniferus*	Orange spiny whitefly	Rose, grape, peach, pear, guava, and citrus	[33]
*Aleurocanthus woglumi*	Citrus blackfly	Lemon, orange, and pomelo.	[34]
*Aleuroclava cardamomi*	Cardamom whitefly	Cardamom	[31]
*Aleurocybotus occiduus*	Rice whitefly	Rice, sorghum and maize	[35]
*Aleurodicus cocois*	Coconut whitefly	Coconut, cashew	[36]
*Aleurodicus disperses*	Spiraling whitefly	Chillies, capsicum, cassava, tomato, eggplant, mulberry, etc.	[37]
*Aleurodicus dugesii*	Giant whitefly	Bamboo, citrus, hibiscus, jasmine, etc.	[38]
*Aleurodicus pseudugesii*	NA	Coconut palm	[39]
*Aleurodicus rugioperculatus*	Rugose spiraling whitefly	Brazilian pepper, mango, palm, and coconut, etc.	[40]
*Aleurodicus talamancensis*	NA	Banana	[41]
*Aleurolobus barodensis*	Sugarcane whitefly	Sugarcane	[42]
*Aleurolobus niloticus*	Nabk whitefly	Nabk	[43]
*Aleurolobus olivinus*	Olive whitefly	Olive	[44]
*Aleuroplatus coronate*	Crown whitefly	Oak, chestnut, etc.	[45]
*Aleurothrixus aepim*	NA	Cassava	[46]
*Aleurothrixus floccosus*	Woolly whitefly	Citrus, cassava, guava, etc.	[47]
*Aleurotrachelus socialis*	Cassava whitefly	Cassava	[48]
*Aleurotrachelu* sp.	Fringed guava whitefly	Guava and kava	[49]
*Aleurotulus anthuricola*	Anthurium whitefly	Anthurium	[50]
*Aleyrodes lonicerae*	Honeysuckle whitefly	Honeysuckle	[51]
*Aleyrodes proletella*	Cabbage whitefly	Cabbage and other brassicas	[52]
*Aleyrodes spiraeoides*	Iris whitefly	Iris, gladiolus, cotton, and potato, etc.	[53]
*Bemisia afer*	Sycamore whitefly	Cotton, cassava	[54]
*Bemisia tabaci* complex	Silverleaf or Sweetpotato whitefly	Cotton, cassava, cucurbits, tomatoes, peppers, brassicas, legumes	[55]
*Bemisia tuberculata*	NA	Cassava	[48]
*Dialeurodes citri*	Citrus whitefly	Citrus, coffee, jasmine, pear, Osage orange, pomegranate, etc.	[56]
*Dialeurodes kirkaldyi*	Jasmine whitefly	Jasmine	[31]
*Dialeuropora decempuncta*	Breadfruit whitefly	Mango, sunflower, cucumber, breadfruit, white mulberry, rose, tomato, etc.	[57]
*Kanakarajiella vulgaris*	Jasmine whitefly	Jasmine	[31]
*Neomaskellia andropogonis*	Sugarcane whitefly	Sugarcane	[58]
*Neomaskellia bergii*	Cane mealy wing whitefly	Sugarcane	[59]
*Orchampoplatus mammaeferus*	Croton whitefly	Garden croton	[60]
*Parabemisia myricae*	Japanese bay berry whitefly	*Citrus* spp. and Gardenia.	[56]
*Paraleyrodes bondari*	Bondar’s nesting whitefly	Citrus, Hibiscus, Ficus, etc.	[61]
*Singhiella cardamomi*	Cardamom whitefly	Cardamom	[31]
*Singhiella citrifolii*	Cloudy winged whitefly	Citrus, Ficusnitida etc.	[62]
*Singhiella pallid*	Betelvine whitefly	Betelvine	[31]
*Singhiella simplex*	Ficus or Fig whitefly	Ficus	[63]
*Siphoninus phillyreae*	Ash whitefly	Pomegranate, plum, peach, citrus, apple, and pear	[64]
*Tetraleurodes mori*	Mulberry whitefly	Citrus, other trees	[45]
*Tetraleurodes ursorum*	Bearberry whitefly	Cassava	[65]
*Trialeurodes abutiloneus*	Banded winged whitefly	Cotton, cucurbits, soybean, brassica, citrus, tomato, beans, eggplant, sweetpotato, etc.	[65]
*Trialeurodes lauri*	NA	Sweet Bay, Grecian strawberry, etc.	[66]
*Trialeurodes manihoti*	NA	Cassava	[46]
*Trialeurodes packardi*	Strawberry whitefly	Strawberry	[67]
*Trialeurodes ricini*	Castor bean whitefly	Castor bean, Indian bean, cotton, pumpkin, sweet potato, tomato, potato, melon, cucumber, okra, and curry plant	[68]
*Trialeurodes vaporariorum*	Greenhouse whitefly	Bean, melon, lettuce, cucumber, tomato, squash, potato, eggplant, strawberry, grape, tobacco, rose, etc.	[69]
*Trialeurodes variabilis*	Cassava whitefly	Cassava and Papaya	[48]

NA = not available.

**Table 3:** Periodic global incidences of whiteflies infestation as well as serious outbreaks of plant diseases vectored by whiteflies on important crops in the last 20 years.

Crop	Year of Whitefly Outbreaks or Recorded Disease Incidences	Disease/Virus/ Whitefly Incidence *	Country/ Territory	Reference(s)
Cotton	2001–2002	*B. tabaci*	Australia	[70]
	2001–2002	CLCuD (100)	Pakistan	[71]
	2002–2003	CLCuD	Pakistan	[72]
	2004–2005	CLCuD (20)	Pakistan	[73]
	2004	CLCuD (up to 100)	India	[74]
	2008	CLCuD (54.24)	Pakistan	[72]
	2009	CLCuD (83.1)	Pakistan	[72]
	2009–2010	CLCuD	India (up to 100% yield loss)	[71]
	2012–2014	CLCuD (37.5 to 63.6)	India	[75]
	2015–2016	Whitefly and CLCuD	India (35% yield loss of worth US$ 630–670 million)	[76,77]
Cucurbits	2000	CYSD (up to 100)	Labanon (40–60% yield reduction)	[78]
	2002–2003	*CVYV*, CYSD	Portugal	[79]
	2006	CYSD	Mexico and USA	[80]
	2007–2009	CYSD (39–100)	USA	[81]
	2007	SLCD	Taiwan	[82]
	2008	CYSD	China	[83]
	2009–2010	*WmCSV* (up to 90), SLCD (up to 100)	Lebanon	[84]
	2011–2012	*CCYV* (up to 49), CYSD (up to 36)	Iran	[85]
	2012–2013	*CCYV*, CYSD (up to 60)	Lebanon	[86]
Tomato	2000	TYLCD (90)	USA (Louisiana, up to 100% yield reduction)	[87]
		TYLCD (15–60)	Greece (loss of US$ 0.5 million)	[88]
		*TICV* (93)	Italy	[89]
	2001	TYLCD (75)	Puerto Rico	[90]
	2001–2002	TYLCD (up to 40)	Tunisia	[91]
	2002–2003	TYLCD (up to 100)	Jordan	[92]
	2003–2004	TYLCD (up to 100)	Italy	[93]
		TYLCD/ToLCD (53)	Mali	[94]
	2002–2004	*ToCV* (31)	France	[95]
	2002–2003/2005	TYLCD (89.19)	Israel	[96]
	2005/2007	TYLCD (88.81)	Lebanon	[96]
	2005/2007	TYLCD (88.61)	Jordan	[96]
	2005/2007	TYLCD (91.25)	Egypt	[96]
	2006	TYLCD (90)	China	[97]
	2006	TYLCD (100)	Australia	[98]
	2007	*TICV*	Jordan	[99]
	2009	TYLCD (up to 50)	Mauritius	[100]
	2009–2012	*TICV* (62.5), *ToCV* (20.5)	Greece	[101]
	2014	*TICV* (100)	Saudi Arabia	[102]
	2014–2016	TYLCD (85)	Trinidad	[103]
	2015–2016	TYLCD	Spain	[104]
	2015–2016	*ToCV* (47)	South Africa	[105]

* Number in parenthesis represents percent disease/viral incidences reported. Note: CLCuD = Cotton Leaf Curl Disease; CYSD = Cucurbit Yellow Stunting Disorder; CuLCrD = Cucurbit Leaf crumple Disease; *CVYV* = *Cucurbit Vein Yellowing Virus*; SLCD = Squash Leaf Curl Disease; *WmCSV* = *Watermelon Chlorotic Stunt Virus*; *CCYV* = *Cucurbit Chlorotic Yellow Virus*; *TICV* = *Tomato Infectious Chlorosis Virus*; *ToCV* = *Tomato Chlorosis Virus*; ToLCD = Tomato Leaf Curl Disease; TYLCD = Tomato Yellow Leaf Curl Disease.

**Table 4:** The implication of RNA interference for the control of sweetpotato whitefly and associated viral diseases.

Sr. No.	Target Genes	Source of Gene Sequence	Target	Function	Developmental Stage Affected	Bioassay Method	Tested Concentration	Significant Effects/Results	Time Span of the Experiment	References
Energy Metabolism										
1	ADP/ATP Translocase	*B. tabaci*	*B. tabaci*	Transmembrane transport	Adults	Artificial diet	20 μg/mL	15% mortality	6 days	[112]
2	Trehalase1	*B. tabaci*	*B. tabaci*	Instant source of energy, role in abiotic stress	Adults	Artificial diet	30 μg/mL	70% mortality	6 days	[120]
3	Trehalose transporter1	*B. tabaci*	*B. tabaci*	Regulation of trehalose levels in the hemolymph	Adults	Artificial diet	30 μg/mL	73% mortality	6 days	[120]
4	ghr-MIR166b	*Gossypium hirsutum*	*B. tabaci*	Regulates the energy metabolism by targeting the ATP synthase gene of *B. tabaci*	Adults	In planta (transgenic tobacco)	NA	78% mortality	15 days	[124]
						Leaf disc	NA	90%	6 days	
**Metamorphosis of Insects**										
5	Cyp315a1	*B. tabaci*	*B. tabaci*	Ecdysone biosynthesis	Adults and 4th instar nymphs	Detached tomato leaf, pre-soaked in a solution of dsRNA	0.5 mg/mL	No significant changes	6 days	[125]
6	Cyp18a1	*B. tabaci*	*B. tabaci*	Ecdysone degradation	Adults and 4th instar nymphs	Detached tomato leaf, pre-soaked in a solution of dsRNA	0.5 mg/mL	No significant changes	6 days	[125]
7	EcR	*B. tabaci*	*B. tabaci*	Ecdysone signaling pathway	Adults and 4th instar nymphs	Detached tomato leaf, pre-soaked in a solution of dsRNA	0.5 mg/mL	Decreased fecundity in adults, mortality in fourth instar nymphs	6 days	[125]
8	E75	*B. tabaci*	*B. tabaci*	Ecdysone signaling pathway	Adults and 4th instar nymphs	Detached tomato leaf, pre-soaked in a solution of dsRNA	0.5 mg/mL	No significant change in adults, mortality in fourth instar nymphs	6 days	[125]
9	Juvenile hormone esterase	*B. tabaci*	*B. tabaci*	Hydrolysis of juvenile hormone	Adults	Artificial diet	2.5 μg/μL	Significant reduction in fecundity and survival of whiteflies	2 days	[122]
**Detoxification Pathway**										
10	P450 CYP6CM1	*B. tabaci*	*B. tabaci* biotype, B and Q	Metabolism of hormones and the catabolism of toxins	Adults	Artificial diet	40 μg/mL	86% and 56% mortality in biotype B and Q, respectively	7 days	[126]
11	GST	*B. tabaci*	*B. tabaci*	Protect cellular macromolecules from harmful xenobiotics	Adults	Artificial diet	1.0 μg/μL	77% mortality	3 days	[127]
					Adults	Artificial diet	1000 mg/L	Increased mortality in thiomethoxam resistant strain	3 days	[128]
					Adults and nymphs	Artificial diet	100 μg/mL	Significantly delayed and reduced progeny emergence, prolonged development period of nymphs	3 days	[121]
					Adults and nymphs	Transgenic *Arabidopsis thaliana*	NA	Significantly delayed and reduced progeny emergence, prolonged development period of nymphs	38 days	
12	AChE	*B. tabaci*	*B. tabaci*	Neuronal transmission and signaling between synapses	Adults	Transgenic tobacco	NA	90% mortality	4 days	[129]
13	BtGSTs5	*B. tabaci*	*B. tabaci*	neutralization of activated glucosinolates		Artificial diet and transgenic *A. thaliana*	100 μg/mL	Plant-mediated dsRNA reduces the insect’s fitness	4 days	[121]
**Insect Immunity and Development**										
14	Toll-like receptor	*B. tabaci*	*B. tabaci*	Larval innate, as well as adaptive immunity	Nymphs	Leaf dipped in the solution of Recombinant *Isaria fumosorosea* strain expressing dsRNA	2 × 10^7^ spores per mL	90.33% mortality of nymphs	12 days	[130]
					Adult	Artificial diet	100 μg/mL DA + 20 μg/mL dsRNA	LC50 of destruxin A and dsRNA = 103.45 μg/mL in comparison to LC50 = 352.7 μg/mL of diet containing DA only	1 days	[123]
15	Defensin-like peptide	*B. tabaci*	*B. tabaci*	Anti-microbial activities against bacteria, fungi and other parasites	Adult	Artificial diet	0.5 μg/μL	Significantly compromised virus carrying capacity of whitefly and density of endosymbiont Rickettsia	2 days	[131]
**Cellular Transport and Osmoregulation**										
16	Aquaporin	*B. tabaci*	*B. tabaci*	Water transport across cell membranes	Adults	Transgenic	NA	78% mortality	6 days	[132]
17	Alpha glucosidase	*B. tabaci*	*B. tabaci*	Osmo regulation	Adults	Transgenic	NA	65% mortality	6 days	[132]
						Artificial diet	30 μg/mL	84% mortality	6 days	[120]
18	Sugar transporters (STs)	*B. tabaci*	*B. tabaci*	Essential for sugar exchange and maintenance of osmotic pressure	Adults	Artificial diet	100 ng/200 μL	Mortality	4 days	[133]
19	V ATPase A	*B. tabaci*	*B. tabaci*	ATP hydrolysis coupled proton transport	Adult	Artificial diet	20 μg/mL dsRNA and siRNA	85.62% in siRNA and 97.5% mortality in dsRNA treatment	6 days	[112]
						Transgenic lettuce	NA	84–98% mortality and 95- fold lower fecundity	5 days	[134]
						Transgenic tobacco	NA	34–83% mortality, respectively	6 days	[113]
**Thermal Tolerance**										
20	HSP 23	*B. tabaci*	*B. tabaci*	Cold acclimation, response to heat	Adults	Artificial diet	0.5 μg/μL	Reduced female survival rate	3 h	[135]
21	HSP 70	*B. tabaci*	*B. tabaci*	Heat shock-mediated polytene chromosome puffing	Adults	Artificial diet	0.5 μg/μL	Reduced female survival rate	3 h	[135]
							250 ng/μL	dsRNA-treated whiteflies lost their vitality and thermal tolerance which leads to increased mortality rate	1 day	[136]
							30 μg/mL	35% mortality	6 days	[120]
22	HSP 90	*B. tabaci*	*B. tabaci*	Response to heat	Adults	Artificial diet	0.3–0.5 μg/μL	No significant changes	1 h and 3 h	[135]
**Embryogenesis and Reproduction**										
23	BtCG5885	*B. tabaci*	*B. tabaci*	Embryogenesis	Adults	Injection	0.1–0.5 μg	Disruption of actin network in developing eggs	2 days	[137]
24	BtGATAd	*B. tabaci*	*B. tabaci*	Embryogenesis	Adults	Injection	0.1–0.5 μg	Disruption of actin network in developing eggs	2 days	[137]
25	Vitellogenin receptor	*B. tabaci*	*B. tabaci*	Uptake of vitellogenin by endocytosis	Adults	Artificial diet	40 μg/mL	Reduction in total egg count, presence of distorted eggs and egg mortality 63.83 ± 6.35%	3 days	[114]
**Cell Division, Shape, Motility, and Intracellular Trafficking**										
26	Alpha tubulin	*B. tabaci*	*B. tabaci*	Essential for fast growth of the microtubules during the initial cleavage divisions of embryogenesis	Adult	Artificial diet	20 μg/mL	34% mortality	3 days	[112]
27	Ribosomal Protein L9	*B. tabaci*	*B. tabaci*	Mitotic spindle elongation; translation; centrosome duplication	Adult	Artificial diet	20 μg/mL	37% mortality	3 days	[112]
28	Actin	*B. tabaci*	*B. tabaci*	Cell mobility	Adults	Artificial diet	20 μg/mL	18% mortality	6 days	[112]
29	BtACTB	*B. tabaci*	*B. tabaci*	Physiological function	Adults	Transgenic tobacco	NA	Reduced survival rate, and impaired fecundity	7 days	[138]
30	Dystrophin	*B. tabaci*	*B. tabaci*	Conserved protein essential for the development of the muscle system	Adults	Roots of tomato dipped into dsRNA solution	0.5 μg/ul	Significant inhibition of the emergence of adults from pupae	23 days	[139]
**Virus Transmission**										
31	Cyclophilin B and hsp 70	*B. tabaci*	*Tomato yellow leaf curl virus* (TYLCV)	Cyclophilin B and hsp 70 interact and co-localize with TYLV in whitefly midgut and help in virus transmission	Adults	Transgenic plant	NA	Whiteflies showed decreased ability to transmit TYLCV under lab conditions	3 days	[140]
32	Odorant-binding proteins (OBPs)	*B. tabaci*	*Tomato chlorosis virus* (ToCV)	OBPs help in identifying plant VOCs in the olfactory recognition of insects	Adults	Artificial diet	500 ng/μL	The viral transmission rate was reduced by 83.3%	40 days	[141]
33	GroEL	*B. tabaci*	*Tomato yellow leaf curl virus* (TYLCV)	Member of chaperonin family helps in virus transmission via binding through coat proteins of the virus	Adults	Transgenic Tomato	NA	Mild or no viral symptoms have been recorded for up to 3 generations of transgenic tomato	2 days	[142]
34	Knot-1	*B. tabaci*	*Tomato yellow leaf curl virus*	Regulates the number of virions in the hemolymph	Adults	Detached tomato leaf, pre-soaked in a solution of dsRNA	0.5 μg/μL	Knot-1 gene silencing leads to a 3-fold increase in the amount of TYLCV acquisition	2 days	[143]
35	Knot -3	*B. tabaci*	*Tomato yellow leaf curl virus*	Regulates the number of virions in the hemolymph	Adults	Detached tomato leaf, pre-soaked in a solution of dsRNA	0.5 μg/μL	No significant effects were observed	2 days	[143]

## Data Availability

Not applicable.
